# Effect of body mass index on pharmacokinetics of paclitaxel in patients with early breast cancer

**DOI:** 10.1002/cam4.3865

**Published:** 2021-04-07

**Authors:** Vikram Gota, Manjunath Nookala, Avinash Bonda, Ashwin Karanam, Bharati Shriyan, Yogesh Kembhavi, Murari Gurjar, Anand Patil, Ashish Singh, Navin Goyal, Sudeep Gupta

**Affiliations:** ^1^ Department of Clinical Pharmacology Advanced Centre for Treatment, Research and Education in Cancer Tata Memorial Centre Navi Mumbai India; ^2^ Homi Bhabha National Institute Mumbai India; ^3^ Department of Medical Oncology Tata Memorial Centre Mumbai India; ^4^ Experimental and Clinical Pharmacology College of Pharmacy University of Minnesota Minneapolis MN USA; ^5^ Clinical Pharmacology GlaxoSmithKline Pennsylvania PA USA

**Keywords:** body surface area, breast cancer, paclitaxel, pharmacokinetics

## Abstract

**Background:**

Paclitaxel is dosed according to body surface area (BSA) but there is scant information on actual drug exposure in overweight and obese patients.

**Methods:**

Early breast cancer patients receiving paclitaxel at 175 mg/m^2^ every 3 weeks, in two BMI groups (normal, 18–24.9 kg/m^2^ and overweight/obese, ≥25 kg/m^2^, respectively), matched for age, serum albumin and bilirubin levels using minimization technique, were included. Sparse pharmacokinetic (PK) sampling was performed at 7 time points from 0 h until 24 h of starting paclitaxel in cycle 1. Paclitaxel concentration was measured using a validated LCMS/MS method. Covariate effect on paclitaxel PK was evaluated by population PK analysis using NONMEM software.

**Results:**

Eighteen female patients each were enrolled in normal and overweight groups with mean BMI of 21.62 ± 2.06 and 28.16 ± 2.31 kg/m^2^, mean BSA of 1.44 ± 0.11 and 1.69 ± 0.14 m^2^ and mean paclitaxel dose of 250 ± 18 and 293 ± 21 mg, respectively. Model predicted AUC and dose normalized AUC (mean ±SD) in the normal BMI versus overweight obese groups were 23 ± 11.0 µmol*h/L versus 25.7 ± 13.7 µmol*h/L (two‐sample *t*‐test *p* > 0.05) and 0.08 ± 0.04 (µmol*h/L)/ µmol versus 0.08 ± 0.04 (µmol*h/L)/ µmol (2‐sample *t*‐test *p* > 0.05), respectively. No significant correlation was observed between BMI and standardized dose normalized AUC (Pearson's correlation coefficient, −0.009; *p* > 0.05).

**Conclusion:**

When dosed according to BSA calculated using actual body weight there is no significant difference in paclitaxel exposure between normal and overweight women. Using alternative descriptors of weight to calculate BSA could lead to under‐dosing of this drug.

**Trial registration:**

This study is registered in the Clinical Trials Registry of India CTRI/2015/09/006193.

## Graphical abstract

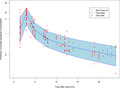

## 1 INTRODUCTION

Determining the optimal starting dose of chemotherapy presents a considerable challenge when using body surface area (BSA) based dosing, particularly in overweight or obese patients.^1^ The paucity of pharmacokinetic data of anti‐cancer drugs in these patients and their under representation in clinical trials have contributed to the dosing uncertainty in this group.^2^ A meta‐analysis involving 213,075 patients from 82 studies showed a 41% increased risk of all‐cause mortality and 35% increased risk of breast cancer‐specific mortality in obese women compared to women with normal body weight.^3^ While multiple factors may have contributed to this detriment, under‐dosing of chemotherapy could be an important contributing factor. Recently, a retrospective analysis of a prospective randomized study in early breast cancer (EBC) showed reduced disease‐free and overall survival with increasing BMI in patients who received docetaxel‐based treatment.^4^ Thus, there continues to be uncertainty about dosing of taxanes in relation to BMI.

Doses of chemotherapy calculated according to BSA tend to be higher in obese patients. Therefore, alternative descriptors of weight to calculate BSA are sometimes used in an attempt to reduce toxicity. Approaches for dose calculation in overweight or obese patients include dose capping, dose banding, flat‐fixed dosing and use of ideal body weight (IBW) rather than actual body weight (ABW).^1^, ^2^ The GAIN study, a large randomized phase III trial which addressed dosing of chemotherapy in obese patients, reported a higher risk of developing severe toxicity in obese patients who were dosed according to their actual BSA. Based on these findings, dose adjustment of dose‐dense chemotherapy was recommended.^5^ On the contrary, a meta‐analysis of 12 studies involving 9314 patients showed similar or lower rates of grade 3/4 hematologic and non‐hematologic toxicities and no difference in progression‐free survival in obese women as compared to normal body weight women when dosed according to actual body weight.^6^

A pharmacokinetic study which evaluated dosing considerations in obese patients reported consistently lower exposures in patients in whom a priori dose reduction or dose capping was used compared with patients who were dosed according to actual body weight.^7^ The American Society of Clinical Oncology (ASCO) recommends full weight‐based chemotherapy doses be used to treat obese or overweight patients with cancer, particularly when the intent is curative.^8^ This recommendation was based on lack of evidence indicating increased incidence of hematological or non‐haematological toxicities in obese patients who received full weight based chemotherapy doses.

Paclitaxel, a semi synthetic compound, is widely used in breast, ovarian, non‐small cell lung and several other cancers. It is highly lipophilic and exhibits non‐linear pharmacokinetics following short infusions and hence concerns remain regarding altered disposition in obese individuals.^9^ Obese individuals tend to exhibit higher absolute drug clearance and volume of distribution (Vd) for this drug.^7^ Very few studies have addressed the issue of paclitaxel pharmacokinetics in relation to body weight. Therefore, we conducted, and report here, a prospective, single‐arm, phase‐II study to evaluate the pharmacokinetic profile of paclitaxel administered according to actual body weight in women with early breast cancer, stratified according to their BMI, using a population pharmacokinetic (popPK) approach.

## 2 MATERIALS AND METHODS

### 2.1 Study design, patients, and setting

This was a single centre, prospective, open label, non‐randomized, pharmacokinetic study which was approved by the Institutional Ethics Committee. Eligibility criteria included women with breast or ovarian cancer undergoing adjuvant or neoadjuvant chemotherapy with paclitaxel at a dose of 175 mg/m^2^ administered as an intravenous infusion over 3 h. Patients were required to have adequate haematological, renal and hepatic functions. Subjects with body surface area more than 2.0 m^2^, BMI of less than 18.5 kg/m^2^, or those who had received whole pelvis radiotherapy were not included in the study. Written informed consent was obtained from all patients prior to study inclusion. The study was carried out in accordance with the Declaration of Helsinki and International Conference on Harmonization—Good Clinical Practice (ICH‐GCP) guidelines.

Patients belonging to two distinct BMI categories described by WHO as ‘normal’ or ‘overweight/obese’ were enrolled. The ‘normal’ cohort comprised of patients with BMI ranging from 18.5 to 24.9 kg/m^2^ while patients with BMI ≥25 kg/m^2^ were classified as ‘overweight/obese’. The two groups were prospectively matched for age (≥60 years and <60 years), serum albumin (≥2.5 mg/dl and <2.5 mg/dl), and serum bilirubin levels (≥0.6 mg/dl and <0.6 mg/dl) which were identified a priori as covariates that could potentially influence the pharmacokinetics of paclitaxel. The covariates were assigned the following codes (A1 –<60 years, A2 – ≥60 years; Al1—serum albumin <2.5 mg/dl, Al2 – ≥2.5 mg/dl; B1—serum bilirubin <0.6 mg/dl, B2 – ≥0.6 mg/dl). Matching was achieved using a minimization strategy to reduce the difference between the two groups with respect to the covariates. Briefly, at the start, five patients were enrolled in each group. Subsequent enrolment depended on whether the next patient minimized the difference between the groups after summing up the number of specific covariates. For example, if the eleventh patient was a 46‐year‐old overweight female with serum albumin level of 2.6 and bilirubin of 0.4, the sum total of A1, Al2, and B1 would be calculated for both groups. The patient would be enrolled only if the difference in the sum of covariates between the groups was reduced.

A sample size of 18 subjects in each arm was required to detect a difference of 20% in the mean area under the curve (AUC) between the two arms with a coefficient of variation of 18%, with power of 90% and alpha error of 5%. Sample size was calculated using G*Power 3.1.5.^10^

### 2.2 Dosing, pharmacokinetic sampling and bioanalysis

Paclitaxel was dosed at 175 mg/m^2^ as a 3‐h infusion, where the BSA was calculated by Mosteller formula.^11^ Standard premedications, including antiemetics, dexamethasone, diphenhydramine, and ranitidine were administered prior to paclitaxel infusion. Pharmacokinetic sampling (3 ml of blood in EDTA tubes) was performed using a sparse sampling strategy immediately pre‐dose, 90 min (mid‐infusion), 180 min (end of infusion), and at 4–5 h, 7–8 h, 10–14 h, and 18–24 h from start of infusion in the first cycle. The time points were identified based on D‐optimal designs for population pharmacokinetic studies. Whole blood collected in EDTA tubes was centrifuged immediately after withdrawal for 10 min at 3000 rpm and the resulting plasma samples were stored at −20°C until bioanalysis. The plasma samples were analyzed for paclitaxel concentrations using a validated LC‐MS/MS method with O‐Methyl paclitaxel as the internal standard as described in previous studies.^12^, ^13^

### 2.3 Pharmacokinetic analysis

Plasma concentration profile of paclitaxel was analysed using both non‐compartmental and population pharmacokinetic analysis. Non‐compartment analysis (NCA) was performed using the NCA plug‐in of Phoenix^®^ WinNonlin^®^ version 7 software (Pharsight Corporation, Mountain View, CA, USA).

Population pharmacokinetics is defined as the study of the sources and correlates of variability in drug concentrations among individuals who are the target patient population receiving clinically relevant doses of a drug of interest. It was performed using NONMEM (Version VI Level 2.0; ICON Development Solutions, Dublin, Ireland), a nonlinear mixed‐effects modelling program.

Data modelling was conducted in two and three compartment models with linear and saturable elimination. Pirana graphic user interface (Version 2.8.1, Pirana Software & Consulting), coupled with PsN (Pearl speaks NONMEM) was used for model building. R coupled to Pirana was used to generate plots. First Order Conditional Estimation with Interaction (FOCE‐I) method was used throughout the analysis. NONMEM performs minimization on the objective function value (OFV), that is, −2 Log Likelihood (−2LL) along with a maximum likelihood approach to arrive at the parameter estimates. Model selection was performed based on the reduction of OFV as calculated by NONMEM, the precision of parameter estimates (relative standard error values) and the diagnostic plots indicating goodness of fit. Between‐subject variability was modelled for V1 and VM assuming a log normal distribution (Equation1):

$${\text{Parameter}}_{i}={\text{Parameter}}_{p}\cdot \exp\left(\eta_{i}\right)$$

where Parameter_i_ is the estimate of a PK parameter of the i^th^ individual, Parameter_p_ is the typical value of the population PK parameter and η_i_ is the estimate of the between subject variability (BSV), normally distributed with a mean of 0 and a variance of ω^2^. Observed concentrations (DV) were used for weighing the residuals to obtain weighted residuals. Residual unexplained variability (RUV) was modelled using a proportional error (Equation 2):

$$C_{i,t}=C_{i,t}^{\prime }\left(1+\varepsilon_{\text{prop},i,t}\right)$$

where C_i,t_ is the observed concentration for ith individual at t time, C’_i,t_ is the individual predicted concentration for ith individual at t time, and ε_prop(i, j)_ is the estimate of RUV for the for ith individual at t time, which is normally distributed with a mean of 0 and a variance of σ^2^. A base model was developed without any covariate effects. Covariate effects such as such as body mass index, body surface area, age, serum albumin, serum globulin, creatinine clearance, and total bilirubin were then tested in a stepwise fashion. First, forward addition followed by, backward deletion. Covariates that upon inclusion reduced the OFV by at least 3.84, (statistical significance of *p* < 0.05, based on a chi‐squared distribution (df =1)) were sequentially added. After stepwise addition of all significant covariates, backward deletion was performed where in removal of covariates that increased the OFV by at least 6.84, (statistical significance of *p* < 0.01, based on a chi‐squared distribution [df = 1]), were considered significant. Additional criteria for evaluating the covariates included were the following: a reduction in unexplained between subject variability, diagnostic plots of the weighted residuals and goodness of fit. Graphic model diagnostics were performed with the following diagnostic plots: individual predicted concentrations (IPRED) versus observed concentrations, and time after dosing versus conditional weighted residuals (CWRES).

Model validation was performed by visual predictive check (VPC). A total of 1000 replicates were simulated using the final model to simulate expected concentrations, and the 90% prediction intervals were generated. The observed data were overlaid on the prediction intervals and compared visually.

### 2.4 Statistical analysis

Covariates could not be modelled in the population PK model because of small sample size and possibility of over‐parametrization. Therefore the area‐under‐the‐curve (AUC) for each individual was calculated using both population pharmacokinetic modelling and NCA. The model AUC and NCA AUC were compared with each other using paired t‐test in order to demonstrate comparability of the estimates using the two methods. These AUCs were then dose normalized to account for different doses prescribed to each subject and then standardized to a mean of zero and standard deviation of one. The standardized dose normalized AUC was correlated with individual BMI and BSA to identify trends using linear fits. Correlation between two continuous measures was determined using Pearson's correlation coefficient. A two‐sample t test was used to compare the means of continuous measures between the normal BMI and overweight/obese groups with a p value of less than 0.05 considered statistically significant.

## 3 RESULTS

### 3.1 Patient characteristics

Between July 2014 and January 2016, 36 adult female subjects were enrolled in the study, 18 each in the normal BMI and overweight/obese cohorts. The median age was 45.5 years with a range of 33–69 years. The BMI ranged from 18.6 to 24.6 kg/m^2^ and 25.6 to 32.5 kg/m^2^ in the normal and overweight/obese cohorts, respectively. Complete patient characteristics are shown in Table 1. A total of 252 samples of plasma for paclitaxel concentrations were obtained from 36 subjects.

**TABLE 1:** Baseline Characteristics

	Total	Normal BMI	Obese BMI
Age (years)			
Median	45	45	46.5
IQR	11.75	5.25	15.5
BMI (kg/m^2^)			
Mean ±SD	24.89 ± 3.95	21.62 ± 2.06	28.16 ± 2.31
ECOG PS			
0	4	0	4
1	32	18	14
BSA (m^2^)			
Mean ±SD	1.56 ± 0.7	1.44 ± 0.11	1.69 ± 0.14
Creatinine Clearance (ml/min)			
Mean ±SD	118.69 ± 31.28	103.83 ± 18.74	133.56 ± 34.58
Weight (kg)			
Mean ±SD	58.53 ± 11.63	49.89 ± 6.61	67.17 ± 8.75
Serum Albumin (g/dl)			
Mean ±SD	4.05 ± 0.29	4.10 ± 0.35	3.99 ± 0.21
Serum Bilirubin (mg/dl)			
Mean ±SD	0.52 ± 0.25	0.52 ± 0.25	0.52 ± 0.25
Stage			
II	8	2	6
III	28	16	12
Paclitaxel dose (mg)			
Mean ±SD	271.64 ± 28.99	250.22 ± 18.05	293.06 ± 20.80

### 3.2 Population pharmacokinetic analysis

**FIGURE 1:**
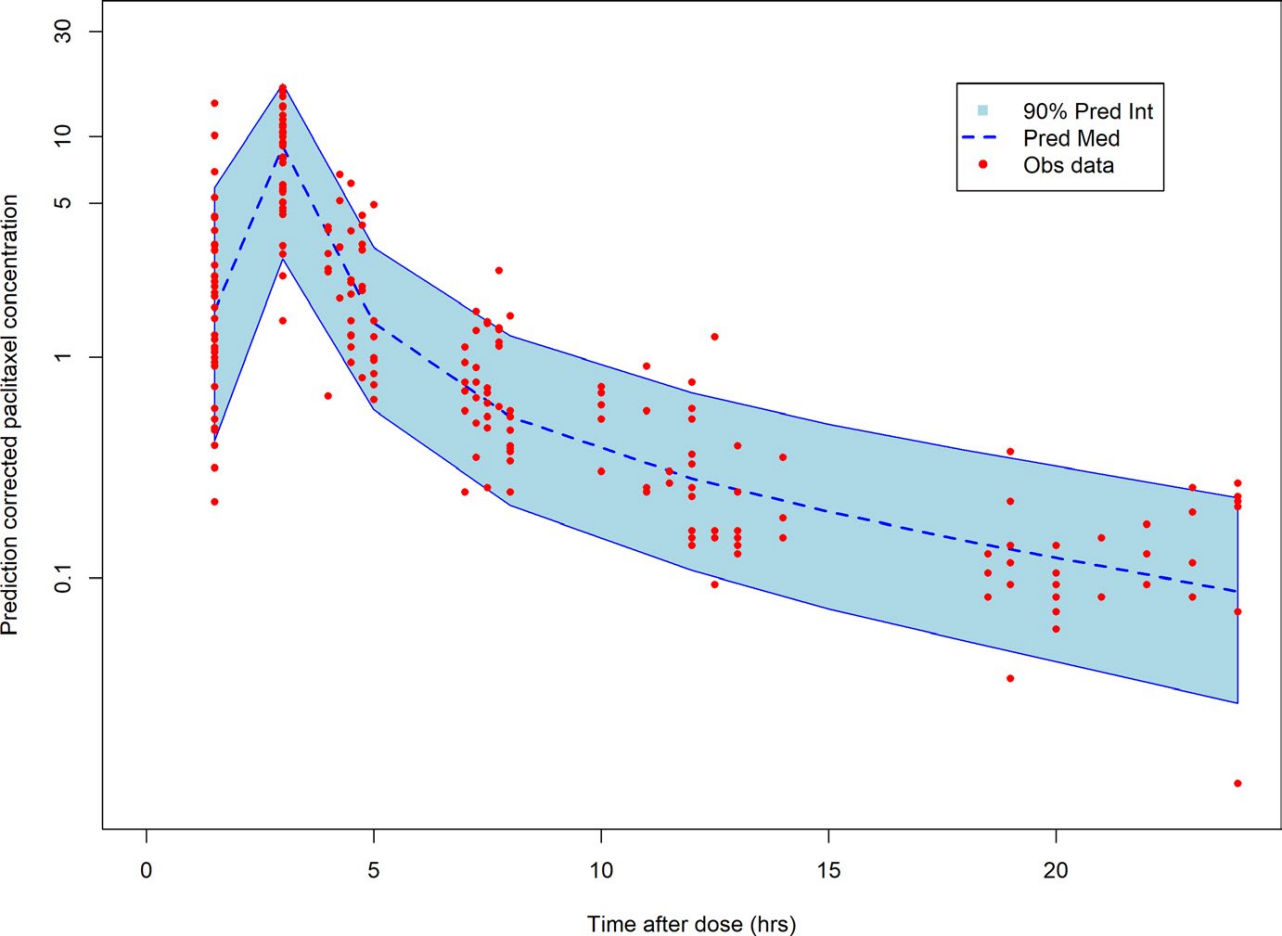
Model Fit: Prediction‐corrected visual predictive check graph, where the observed concentrations (circles) are within 90% of the model predicted quantiles (shaded area) showing adequacy of model fit

A three compartment, mixture model with non‐linear elimination and saturable distribution to one peripheral compartment model was selected as the final model. The code used in the final model is given in Appendix S1. Because we had only 7 plasma paclitaxel concentrations per subject and addition of any more parameters to this model would lead to over‐parametrization, no covariates were tested for significance in this model. However, even with no covariates added, our model was able to describe the data well. The goodness of fit plots are shown in Figure S1. A summary of the population estimates of the paclitaxel are described in Table 2. It is interesting to note that we found two patient populations for volume of central compartment (V1) with 17.3% of the population having a V1 of 23.9 L and the remaining having a V1 of 5.35 L. Upon further analysis, we did not find any correlations between these two sub‐populations and weight, BMI or BSA. The model predicted the elimination Michaelis–Menten constant (KM elimination) to be 0.412 µmol/L, and the distribution Michaelis–Menten constant (KM distribution) to be 0.216 µmol/L indicating that the saturation occurs at therapeutic levels, which further supports use of a non‐linear elimination and distribution. The visual predictive check (VPC) demonstrating model performance is shown in Figure 1. The VPC plot shows that most of the observed values fall within 90% of the predicted values.

**TABLE 2:** Results of the population pharmacokinetic analysis

Parameter	Estimate	BSV (%)	RSE (%)
V1: Population 1 (L)	23.9	—	22
V1: Population 2 (L)	5.35	—	9
Population with V1 (% of total population)	17.3	—	44
VM Elimination (µmol/L/h)	26.7	29	26
KM Elimination (µmol/L)	0.412	—	54
VM Distribution (µmol/L/h)	569	25	67
KM Distribution (µmol/L)	0.216	—	24
V3 (L)	43.4	—	26
K21 (/h)	3.8	29.5	70
Q3 (ml/h)	5.59	—	12
Residual Error (%)	29	—	6

Abbreviations: BSV, Between Subject Variability; K21, Rate constant from the first peripheral compartment to the central compartment; KM, Plasma concentration at half VM; Q, Intercompartmental clearance between the central and second peripheral compartment; RSE, Relative Standard Error; V1, Volume of the central compartment; V3, Volume of the second peripheral compartment; VM, Maximal elimination rate.

### 3.3 Effect of BMI on paclitaxel pharmacokinetics

**FIGURE 2:**
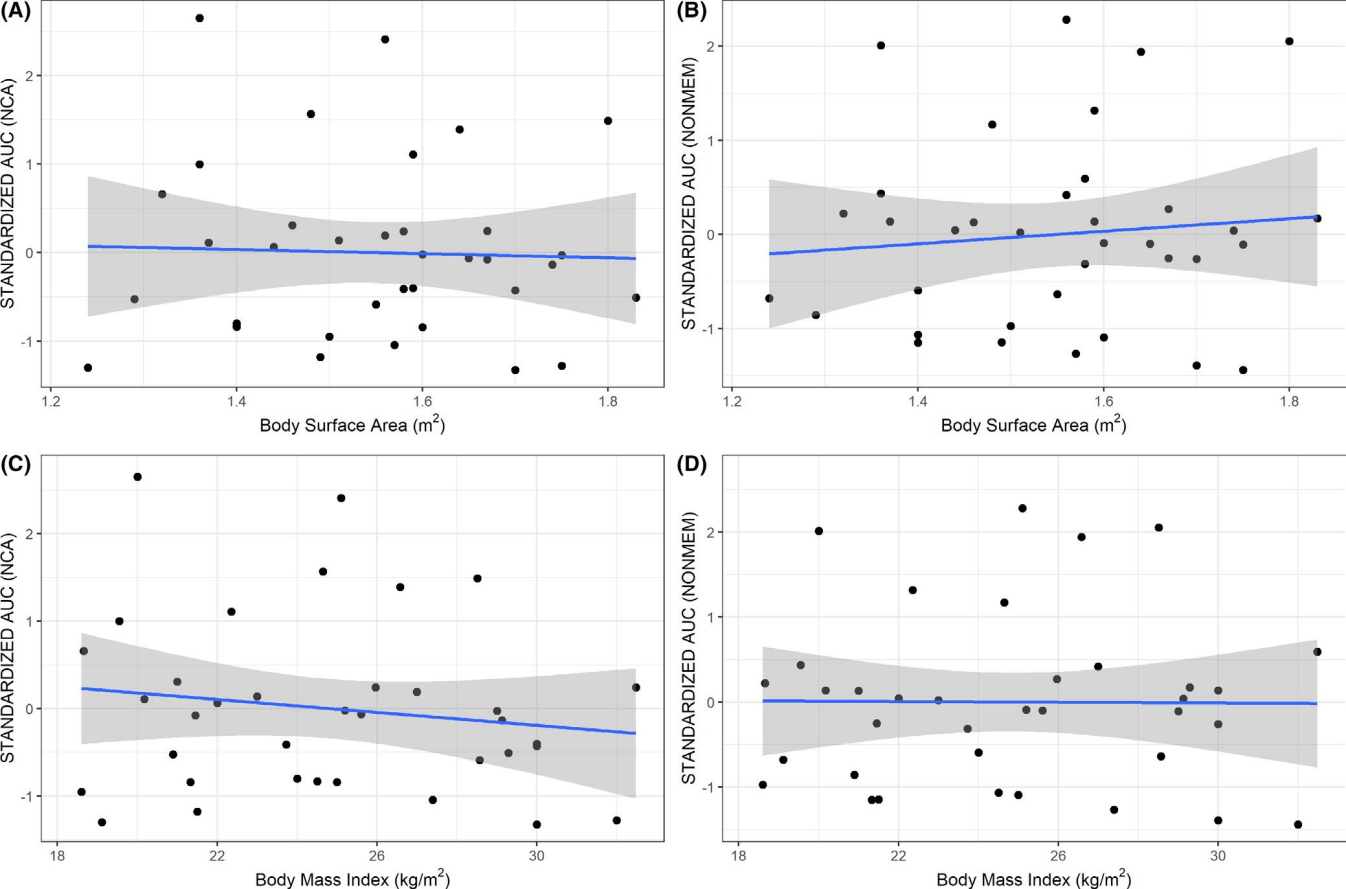
Effect of BMI and BSA on AUC: Plots of body mass index (BMI) and body surface area (BSA) versus standardized dose normalized AUC. A. BSA versus AUC calculated by non‐compartment analysis; B. BSA versus AUC calculated by population pharmacokinetic analysis; C. BMI versus AUC calculated by non‐compartment analysis; D. BMI versus AUC calculated by population pharmacokinetic analysis. The blue lines are linear trend lines, with the shaded area representing the standard error of regression

**FIGURE 3:**
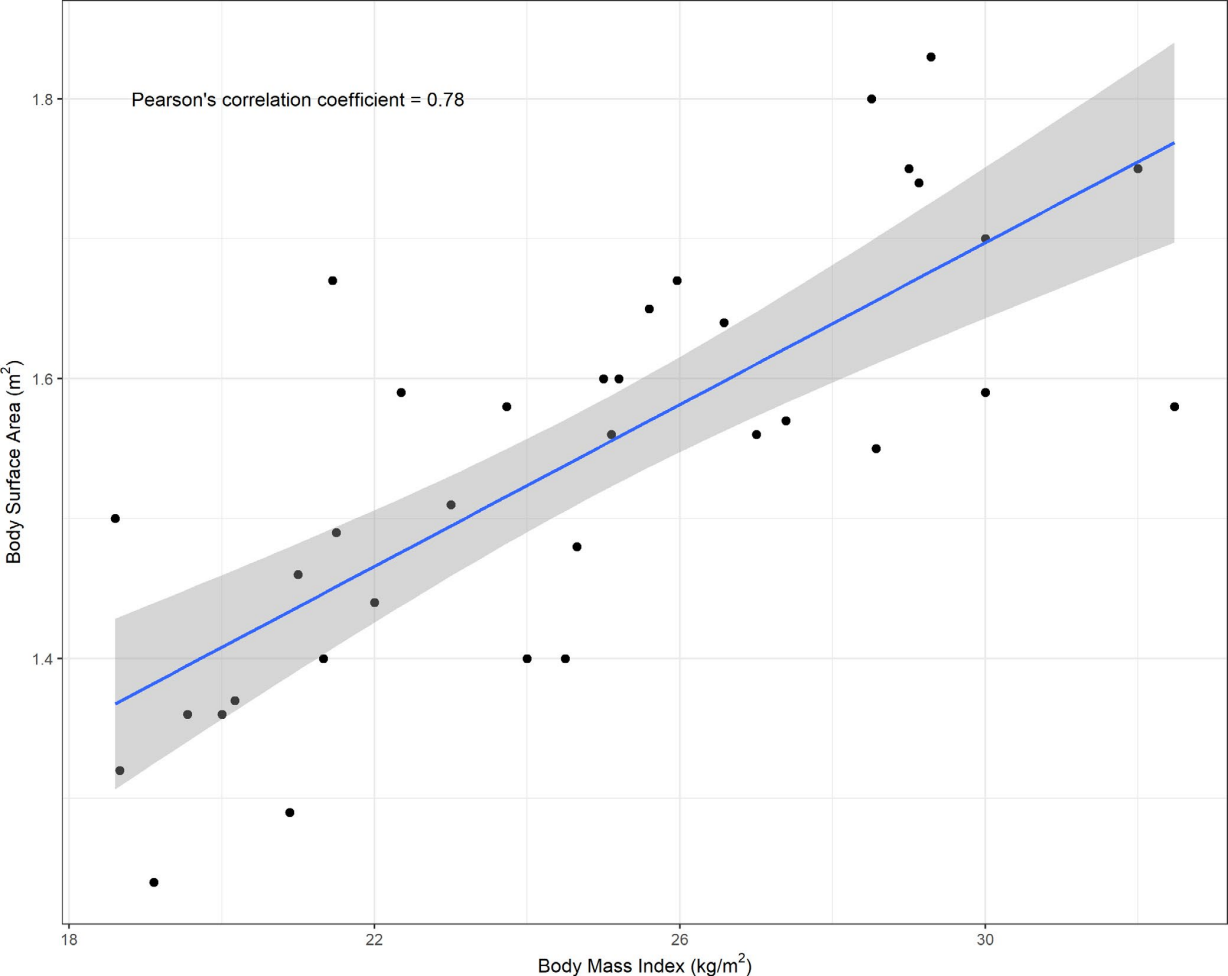
Correlation of BMI and BSA: Plot of body mass index versus body surface area. The blue line is the linear regression line for the plot and the shaded region is the standard error for the regression line

Model AUC (mean: 24.6 µmol*h/L; standard deviation: 12.3 µmol*h/L) and NCA AUC (mean: 26.7 µmol*h/L; standard deviation:14.7 µmol*hr/L) were found to be comparable (paired *t*‐test *p* > 0.05). The model predicted AUC (mean ±standard deviation) in the normal BMI (23 ± 11.0 µmol*h/L) and overweight/obese (25.7 ± 13.7 µmol*h/L) groups were not significantly different (two sample *t*‐test *p* > 0.05). Dose normalized model predicted AUC (mean ±standard deviation) in the normal BMI (0.08 ± 0.04 [µmol*h/L]/µmol) and overweight/obese BMI (0.08 ± 0.04 [µmol*h/L]/µmol) groups were also not significantly different (two sample t‐test *p* > 0.05). Figure 2 shows scatter plots of BMI and BSA versus standardized dose normalized AUC using both NCA and model based AUC. Linear fits show that there is no significant correlation between the NCA‐AUC (Pearson's correlation coefficient −0.03, *p* > 0.05) or model‐AUC (Pearson's correlation coefficient =0.1, *p* > 0.05) and BSA. Linear fits also showed no significant correlation between the NCA‐AUC (Pearson's correlation coefficient −0.014, *p* > 0.05) or model‐AUC (Pearson's correlation coefficient −0.009, *p* > 0.05) and BMI Clearly, BMI and BSA had no effect on paclitaxel exposure. Furthermore, when we looked at the relationship between BMI and BSA, structural collinearity was observed (Pearson's correlation coefficient 0.78, *p* < 0.05) between them (Figure 3).

## 4 DISCUSSION

Our results suggest that pharmacokinetics of paclitaxel in female patients with early breast cancer, dosed according to BSA based on actual body weight, is well described by a three‐compartment model with non‐linear distribution and elimination. Our model estimates are comparable with those reported by others using similar models.^14^, ^15^, ^16^ Dose normalized paclitaxel AUC, estimated by non‐compartmental analysis and NONMEM, were not significantly influenced by either body mass index or body surface area. Of note, we matched the normal BMI and overweight/obese BMI groups for age, serum albumin, and serum bilirubin, which have been reported in some studies to influence the pharmacokinetics of paclitaxel.^14^, ^17^

The findings of this study indicate that BMI does not influence exposure (AUC) to paclitaxel as long as the drug is dosed according to BSA calculated using actual body weight. This is in concordance with the observations made in several other studies wherein no differences in toxicity or survival were observed when chemotherapeutic agents, including paclitaxel, were dosed as per actual body weight^17^, ^18^ and support the recommendations of the ASCO panel pertaining to chemotherapy dosing in obese or overweight patients.^7^ Our findings provide a pharmacokinetic basis for optimal dosing of paclitaxel in obese or overweight patients receiving adjuvant chemotherapy for early breast cancer. Although the study was conducted in patients with early breast cancer, the findings are likely to be applicable to all other indications of paclitaxel, as long as correlation between BMI and BSA exists.

An important implication of our results is that alternative dosing schemes of paclitaxel like dose capping, use of ideal (rather than actual) body weight to calculate BSA and fixed dose regimens may lead to under exposure to this drug in many patients.

This study has some limitations. There was over‐parameterisation of the model due to lack of intensive data‐points, leading to high error in parameter estimates, even though the estimates were similar to literature data and the model was able to describe the observed concentrations well. We did not correlate paclitaxel exposure with either paclitaxel toxicity or efficacy which could have provided important additional insights.

To conclude, our study provides a strong pharmacokinetic basis for dosing paclitaxel according to body surface area calculated using actual body weight in overweight or obese patients with early breast cancer.

## CONFLICT OF INTEREST

None.

## Lay summary

Paclitaxel is an important drug used in the treatment of early stage breast cancer. The dose of paclitaxel is calculated according to body surface area (BSA). However, in women who are overweight or obese, there is a perceived higher risk of toxicity when full dose based on BSA is administered. In this study, we have shown through the measurement of paclitaxel levels in blood, that overweight women achieve comparable levels of paclitaxel as their normal counterparts when administered the full dose. Thus reduced doses based on alternative methods of calculation should be avoided.

## Precis for use in the Table of Contents

This study in early breast cancer patients showed no significant difference in paclitaxel exposure between normal and overweight/obese women when dosed according to BSA calculated using actual body weight. Using alternative descriptors of weight to calculate BSA could lead to under dosing of the drug.

## Data Archiving Statement

Raw data generated in this study is available with the Tata Memorial Centre Research Administration Council.

## Supporting information

Appendix S1‐Fig S1

## Data Availability

The data that support the findings of this study are available from the corresponding author upon reasonable request.
