# Supplementary material for: Effect of body mass index on pharmacokinetics of paclitaxel in patients with early breast cancer
Source: Cancer Med. 2021 Apr 7;10(9):3068–76. doi: 10.1002/cam4.3865 (PMC8086018; doi:10.1002/cam4.3865)
Supplement: Supplementary file 1 — Appendix S1‐Fig S1 [file CAM4-10-3068-s001.docx]

**Supplementary Appendix: Effect of body mass index on pharmacokinetics of paclitaxel in patients with early breast cancer**

1. **The model code used in the analysis**

;; 1. Based on: run575

;; 2. Description: Final paclitaxel model

;; x1. Author: Ashwin

$PROBLEM 3 cmt nl elimination

$INPUT ID TIME DV LNDV AMT RATE BMI AGE BSA LBW IBW ABW ALB BIL WT EVID DOSE

$DATA molconpax.csv IGNORE=@

$SUBROUTINES ADVAN6 TOL=5

$MODEL

COMP(CENTRAL DEFOBS)

COMP(PERI1)

COMP(PERI2)

COMP(AUC)

$PK

VMELPOP=THETA(2)*(WT/70)**0.75

VMEL=VMELPOP*EXP(ETA(1))

KMEL=THETA(3)

VMT=THETA(4)*EXP(ETA(2))

KMT=THETA(5)

V3=THETA(6)

K21=THETA(7)*EXP(ETA(3))

Q3=THETA(8)

R1=RATE

W =THETA(9)

IF (MIXNUM.EQ.1) THEN

V1POP = THETA(1)

ELSE

V1POP = THETA(10)

ENDIF

V1P=V1POP

V1=V1P

;REPARAMATERIZATION

K13 = Q3/V1

K31 = Q3/V3

$MIX

NSPOP=2

P(1) = THETA(11)

P(2)=1-P(1)

$DES

DC1=A(1)/V1

DADT(1) = K21*A(2)+K31*A(3)-K13*A(1)-DC1*VMT/(KMT+DC1)-DC1*VMEL/(KMEL+DC1)

DADT(2) = DC1*VMT/(KMT+DC1)-K21*A(2)

DADT(3) = K13*A(1)-K31*A(3)

DADT(4) = A(1)/V1

$ERROR

IPRED = A(1)/V1

Y=IPRED*(1+EPS(1)*W)

IRES=DV-IPRED

IWRES=IRES/DV

AUC=A(4)

$THETA (0,10,50);V1POP1

$THETA (0,25);VMEL

$THETA (0,1.51);KMEL

$THETA (1,100);VMT

$THETA (0,0.5);KMT

$THETA (0,150);V3

$THETA (0,1.41);K21

$THETA (0,20);Q3

$THETA (0.1);SIGMA

$THETA (0,2);V1POP2

$THETA (0,0.2,1);NSPOP

$OMEGA

0.2;BSV VMAX

0.2;BSV VMT

0.2;BSV K21

$SIGMA

1 FIX; RUVp

$EST METHOD=1 INTERACTION MAXEVAL=9999 SIG=3 PRINT=5 NOABORT POSTHOC

$COV PRINT=E

$TABLE ID TIME DV IPRED IRES ETA1 ETA2 ETA3 BMI BSA EVID W CWRES ONEHEADER NOPRINT FILE=sdtab576

$TABLE ID V1 V3 KMEL VMEL VMT KMT K21 Q3 BMI BSA AUC ETA1 ETA2 ETA3 ONEHEADER FIRSTONLY NOPRINT FILE=patab576

1. **The Goodness-of-Fit Plots**

**Figure S1** showing goodness of fit plots. Top panel: Individual predicted vs observed concentration (left) showing marked better fit compared to population predicted vs observed concentration (right). The black line shows line of identity and the red line shows a local regression line of the data. Bottom panel: population predicted concentrations vs conditional weighted residuals (left) and time after dose vs conditional weighted residuals (right) showing no bias in the error models.
